# *De novo* assembly of middle-sized genome using MinION and Illumina sequencers

**DOI:** 10.1186/s12864-018-5067-1

**Published:** 2018-09-24

**Authors:** Ryuhei Minei, Ryo Hoshina, Atsushi Ogura

**Affiliations:** Department of BioScience, Nagahama Institute of Bio-Science and Technology, Tamura 1266, Nagahama, Shiga 526-0829 Japan

**Keywords:** Plastid acquisition, Secondary endosymbiosis, *Chlorella variabilis*, Genome assembly pipeline, Hybrid assembly, Gene model quality, RNA-seq mapping rate

## Abstract

**Background:**

The plastid acquisition by secondary endosymbiosis is a driving force for the algal evolution, and the comparative genomics was required to examine the genomic change of symbiont. Therefore, we established a pipeline of a de novo assembly of middle-sized genomes at a low cost and with high quality using long and short reads.

**Results:**

We sequenced symbiotic algae *Chlorella variabilis* using Oxfofrd Nanopore MinION as the long-read sequencer and Illumina HiSeq 4000 as the short-read sequencer and then assembled the genomes under various conditions. Subsequently, we evaluated these assemblies by the gene model quality and RNA-seq mapping rate. We found that long-read only assembly could not be suitable for the comparative genomics studies, but with short reads, we could obtain the acceptable assembly. On the basis of this result, we established the pipeline of de novo assembly for middle-sized algal genome using MinION.

**Conclusions:**

The genomic change during the early stages of plastid acquisition can now be revealed by sequencing and comparing many algal genomes. Moreover, this pipeline offers a solution for the assembly of various middle-sized eukaryotic genomes with high-quality and ease.

**Electronic supplementary material:**

The online version of this article (10.1186/s12864-018-5067-1) contains supplementary material, which is available to authorized users.

## Background

Evolutionary research has revealed divergence in photosynthetic eukaryotes with plastid acquisition by secondary endosymbiosis as the driving force [1–3]. This phenomenon has occurred many times over the course of evolution. Plastid acquisition by secondary endosymbiosis consists of four stages: In the first stage, host organisms prey on algae, and undigested algae temporarily become symbionts. In the second stage, the temporary symbionts become persistent symbionts. In the third stage, horizontal gene transfer from the symbiont to the host nucleus occurs. In the final stage, the nucleus of the symbiont disappears, resulting in the establishment of a plastid [4]. Curtis et al. [5] previously studied the third stage of plastid acquisition by genome and transcriptome analysis of cryptophytes and chlorarachniophytes. However, few studies have focused on the early stages of plastid acquisition.

The symbiotic relationship between the ciliate *Paramecium bursaria* and the symbiotic green algae *Chlorella variabilis* is considered a model of the second stage of plastid acquisition [6]. Blanc et al. [7] sequenced the genome of *C. variabilis* to study the genetic basis of symbiosis. However, as genome data were not available for symbionts other than *C. variabilis* and closely related species, it was not possible to compare genomes to clarify the genomic changes associated with becoming a symbiont. To shed light on the genomic change of the early stages of plastid acquisition, it is necessary to sequence the genomes of symbionts and free-living species other than *C. variabilis*; there are many species of symbiotic green algae, and the host organisms have diversified to Ciliates (Alveolata) as well as Amoebozoa, Heliozoa, and other protists [8]. Thus, we must examine genomic changes in these symbionts and the transcriptomic interactions of hosts and symbionts. High-quality assemblies of multiple isolates of both free-living and symbiotic Chlorella are needed to address the second stage of plastid acquisition. For this purpose, it is necessary to assemble many middle-sized (30~ 60 megabase pairs) algal genomes for detailed analysis, which requires a low cost and high-quality method [9–11].

For high-quality genome assembly that reflects gene content and genome structure, long-read sequencers are preferred to short-read sequencers. Read lengths from short-read sequencers are 50–400 bp, resulting in highly fragmented genome assemblies. By contrast, long-read sequencers can sequence long-repeated regions and copy number variations, providing reads up to hundreds of kilobases (kbp) in length [12]. However, long-read sequencers such as PacBio RS2 and Sequel have high costs [13]. They are not suitable for sequencing many green algae genomes. Therefore, we chose the Oxford Nanopore MinION long-read sequencer, which has been distributed since 2014, for this work. MinION has five advantages: the low cost of sequencing, ultra-long reads up to 200 kbp, portability, real-time analysis, and direct molecule analysis [12]. For these reasons, MinION has the potential to revolutionize genomic research. However, there are few reports validating de novo assembly of middle-sized genomes using MinION.

**Fig. 1:**
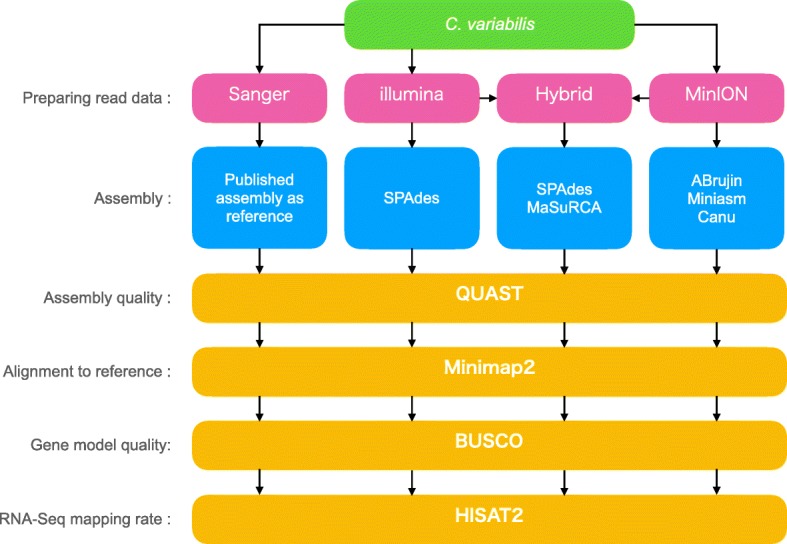
Overview of the workflow for comparing assemblies under various conditions. First, genomic DNA extraction of *C. variabilis* is shown in green. Sanger, Illumina, and MinION indicates sequence reads data derived from these sequencers in the second step (pink). Hybrid indicates mixed data of Illumina and MinION. In the third step (blue), genome assembly under various conditions was performed, and each term shows the name of assembler software. Assembly using reads derived from Sanger sequencer was taken from previously published data [7]. The next steps (yellow) are evaluation of assemblies, each term shows the name of software. The alignment to reference indicates result of aligning each assembly to reference

Here, we aimed to establish a pipeline of de novo assembly for middle-sized genomes at a low cost and with high-quality using MinION for multiple green algal species. We sequenced *C. variabilis* using MinION as the long-read sequencer and Illumina HiSeq 4000 as the short-read sequencer and then assembled the genomes under various conditions (Fig. 1). Subsequently, we evaluated these assemblies by assessing whether contigs were successfully connected to each other (assembly quality). We also compared these assemblies to a high-quality reference previously assembled by Blanc et al. [7] using Sanger sequencing. Lastly, we examined if these assemblies could be used for actual comparative genomics using the gene model quality and RNA-seq mapping rate.

## Results and discussion

### Comparison of assemblies under various conditions

Sequencing *C. variabilis* using MinION and HiSeq generated 78X depth short reads and 56X depth of 578,473 bp long reads. In previous studies, the *Escherichia coli* genome was assembled using 20X MinION reads [14], and the *Saccharomyces cerevisiae* genome was assembled using 30X MinION reads [15]. A depth of 56X is considered a sufficient amount of sequence reads to assemble the chlorella genome. Regarding sequenced raw reads from MinION, we obtained relatively long reads, with an average length of 4.45 kbp, maximum read length of 766,552 bp, and sequencing accuracy of 87.4% (Additional file 1: Figure S1 and Additional file 2: Table S1).

Using these reads, we assembled genomes under various conditions. We assembled genome using only short reads by SPAdes [16] assembler (short-read assembly). Using only long reads, we assembled genomes using three assemblers, namely, ABrujin [17] (ABrujin-assembly), Miniasm [18] (Miniasm-assembly), and Canu [19] (Canu-assembly), which are commonly used in published papers. In addition, we tested the hybrid genome assembly of long and short reads using the SPAdes [14] (SPAdes-hybrid-assembly) and MaSuRCA assemblers [20] (MaSuRCA-assembly). We evaluated assemblies by assembly quality, the coverage, and percent sequence identity by aligning each assembly to the reference. The quality of the reference assembly using Sanger sequencing was relatively good: the number of contigs was small, and the largest contig and N50 were long, as it was assembled using relatively long reads (400~ 900 bp). However, this reference has two problems. First, the number of Ns per 100 kbp was large (8547) and inserted upon scaffolding contigs, preventing precise gene model assumptions. Second, most of the contigs were very small even though the largest contig and N50 were long (Table 1).

**Table 1:** Summary of evaluation for assembled genomes of *C. variabilis* under various conditions using various indexes

	Reference	Short-read	Long				Hybrid	
			ABrujin	Abrujin-polished	Miniasm	Canu	SPAdes-hybrid	MaSuRCA
Assembly quality								
# contigs (> = 0 bp)	414	13,015	259	170	492	2400	10,635	302
# contigs (> = 1000 bp)	414	1870	259	170	492	2399	1079	302
# contigs (> = 5000 bp)	134	1171	259	170	484	1909	772	241
# contigs (> = 10,000 bp)	82	950	259	170	438	1158	664	196
# contigs (> = 25,000 bp)	55	642	259	170	335	141	511	150
# contigs (> = 50,000 bp)	44	348	225	164	243	18	359	134
Total length (> = 0 bp)	46,159,515	58,108,416	44,173,773	45,397,519	42,468,310	27,800,588	58,637,084	46,674,734
Total length (> = 1000 bp)	46,159,515	56,312,404	44,173,773	45,397,519	42,468,310	27,799,589	57,237,973	46,674,734
Total length (> = 5000 bp)	45,602,804	54,680,763	44,173,773	45,397,519	42,437,055	26,338,534	56,523,201	46,494,724
Total length (> = 10,000 bp)	45,222,671	53,103,981	44,173,773	45,397,519	42,054,109	20,603,401	55,758,312	46,162,375
Total length (> = 25,000 bp)	44,846,071	48,059,147	44,173,773	45,397,519	40,395,860	5,591,416	53,252,484	45,478,395
Total length (> = 50,000 bp)	44,435,177	37,767,367	42,798,957	45,170,192	36,950,227	1,766,192	47,753,927	44,916,352
# contigs	414	2540	259	170	492	2400	1479	302
Largest contig	3,119,887	765,833	1,514,322	1,157,783	685,202	327,336	770,020	2,552,940
Total length	46,159,515	56,771,972	44,173,773	45,397,519	42,468,310	27,800,588	57,502,328	46,674,734
GC (%)	67.1	67.9	64.4	65.8	64.8	62.2	67.8	67.1
N50	1,469,606	77,546	250,313	376,772	161,216	14,421	130,737	501,441
N75	953,202	36,956	137,022	228,146	84,461	9866	66,441	250,951
L50	12	195	54	35	77	599	127	23
L75	21	462	113	75	166	1183	279	55
# N’s per 100 kbp	8547	27	0	0	0	0	139	13
Alignment to reference								
Coverage	–	92.21%	94.68%	99.73%	90.23%	26.09%	93.76%	100.77%
Identity	–	89.19%	21.16%	69.82%	14.69%	2.56%	89.96%	97.23%
Gene model quality								
ECR	82.90%	86.50%	1.30%	10.90%	0.00%	0.00%	86.80%	88.50%
RNA-seq Mapping Rate	90.32%	95.81%	23.08%	56.81%	18.68%	5.74%	96.25%	95.06%

Statistics of assembly quality are based on contigs of size > = 500 bp, unless otherwise noted (e.g., “# contigs (> = 0 bp)” and “Total length (> = 0 bp)” include all contigs). Identity indicats the percent sequence identity to the reference

Comparing the short assembly with the reference, the number of contigs was large and the largest contig and N50 were short, indicating difficulty in scaffolding from short reads only. Additionally, the short-read-assembly had 14,208,652 bp that did not align to reference, suggesting that the assembly using only short reads contains many inaccurate sequences (Table 1). In assemblies using only long reads, ABrujin-assembly, and Miniasm-assembly covered almost the whole genome of *C. variabilis* as the coverage rates exceeded 90%. ABrujin-assembly had the highest assembly quality among assemblies using only long reads. In particular, ABrujin-assembly was able to assemble the reads into a small number of contigs than the reference. Although the largest contig and N50 in ABrujin-assembly were shorter than in the reference, the number of contigs over 50 kbp (225) was larger and each contig was longer on average, suggesting that assembly using long reads is suitable for analysis of clustered genes and synteny. Conversely, the Canu-assembly did not cover the whole genome of *C. variabilis* and the total size of the genome (27,800,588 bp) was much smaller than of the reference (about 46 Mbp), and had remarkably lower indices of assembly quality than those for the other assemblies, suggesting that this assembler is not suitable for assembling the *C. variabilis* genome using long reads only (Table 1). Regarding hybrid assembly, SPAdes-hybrid-assembly and MaSuRCA-assembly had a higher coverage and percent sequence identity to the reference than the short-read-assembly. However, SPAdes-hybrid-assembly gave the assembly quality as low as that of short-read-assembly, since scaffolding using long reads was insufficient. On the other hand, the MaSuRCA-assembly resulted in a smaller number of contigs than the reference, a larger contig and N50 than the ABrujin-assembly, and the highest assembly quality. The values of the MaSuRCA-assembly were the highest among our results. In summary, the percent sequence identity to the reference using long reads was lower than that using short-read-assembly or assemblies using only short reads, as the sequencing accuracy of MinION was low. By contrast, hybrid assembly showed improved results as the accuracy of short reads could correct the sequences from MinION.

We assumed that a high-quality assembly could be obtained using only long reads from MinION if the sequencing errors were improved, as the assembly quality and coverage of the ABrujin-assembly were high. Consequently, we polished ABrujin-assembly (ABrujin-polished-assembly), which is the highest evaluated assembly among the long-read assemblies, using Racon [21] and Nanopolish [22]. As a result, the percent sequence identity to the reference for the ABrujin-assembly improved from 22.16 to 69.82% (Table 1). However, the improved percent sequence identity was still lower than that for short-read-assembly.

### Gene model quality

**Fig. 2:**
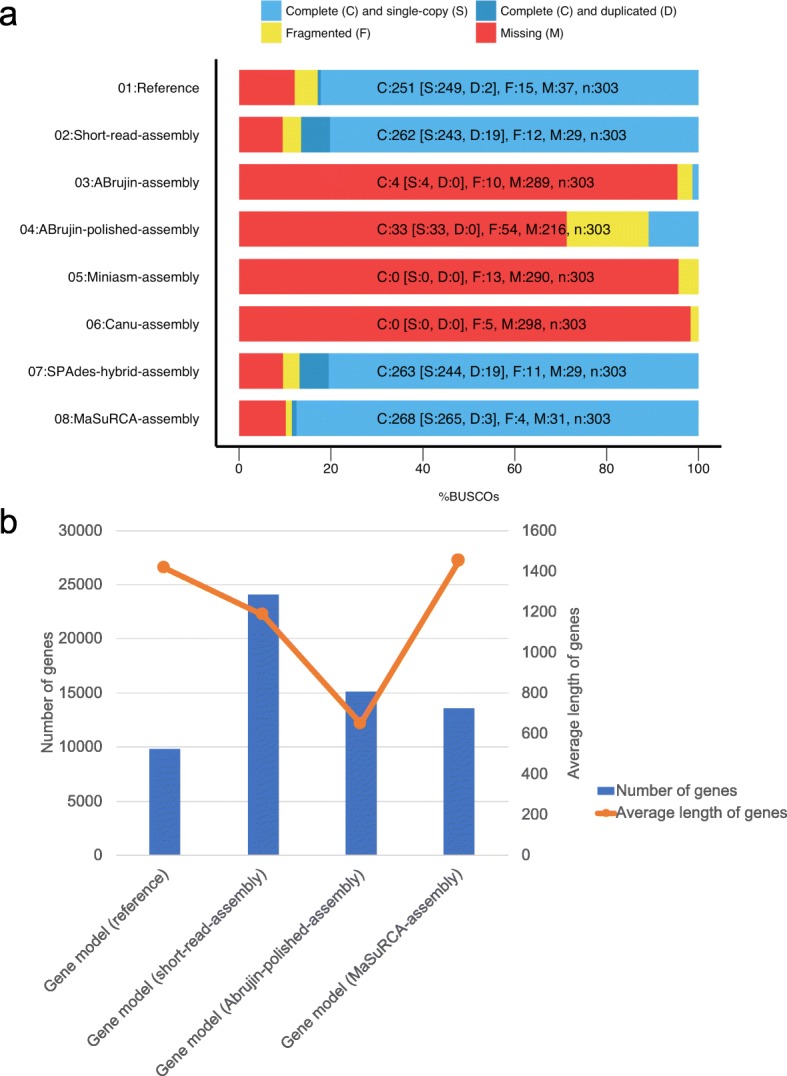
Evaluation of assemblies using gene model quality. **a** Assessment of ECR on eight assemblies using BUSCO. The x-axis represents of ECR patterns. **b** Evaluation of estimated four gene models of four assemblies using average length of genes (orange line) and the number of genes (blue bar).

Precise prediction of genes is essential for comparative genomics to reveal the common process in the second stage of plastid acquisition related to gene duplication, gene loss, and horizontal gene transfer. Therefore, we evaluated the precision of gene recovered in the assemblies constructed above by the common eukaryotic genes conservation rate (ECR) using BUSCO [23]. ECR is defined as the proportion of 303 common eukaryotic single-copy-orthologs (core genes) recovered in the genome assemblies in comparison [23]. The ECR of the reference, short-read-assembly, ABrujin-assembly, ABrujin-polished-assembly, Miniasm-assembly, Canu-assembly, SPAdes-hybrid-assembly, and MaSuRCA-assembly were 82.90%, 88.50%, 1.30%, 10.90%, 0.00%, 0.00%, 86.80%, and 88.50%, respectively (Table 1). The reason why the ECR of the short-read-assembly and SPAdes-hybrid-assembly was higher than that of reference is that these assemblies had 19 duplicated core genes (Fig. 2a). These duplications are artifacts of low assembly quality. The ECRs were quite low for assemblies using only long reads, as the sequencing accuracy of MinION is low. Though its percent sequence identity was remarkably improved by polishing, the ECR of ABrujin-polished-assembly was still extremely low. This indicates that the ABrujin-polished-assembly is not useful for actual genome analysis. The ECR of the MaSuRCA-assembly was the highest of all assemblies including the reference, and core gene duplication was not observed.

Next, we performed whole-gene model prediction using the short-read-assembly, ABrujin-polished-assembly and MaSuRCA-assembly, and evaluated the number and average length of the gene model to compare these gene models to the reference gene model previously proposed [7]. In the gene model for the short-read-assembly, the number of genes was more than double that for the reference gene model, and the average length of genes was different from that for the reference gene model. In the gene model for the ABrujin-polished-assembly, the average length of genes was dramatically lower than for the other models, indicating that the ABrujin-polished-assembly model is not suitable for genome analysis because start and stop codons were incorrectly introduced in the middle of the coding sequence in this model.

### Evaluation of RNA-seq mapping rate

Gene expression analysis can provide essential clues to help us understand how hosts and symbiont live together at the second stage of plastid acquisition. This analyses should be processed by mapping RNA-seq to the assembled genome, not by de novo assembly of RNA-seq, as it is crucial to identify the origin of the highly conserved genes. We evaluated whether reliable gene expression analysis could be performed by calculating the RNA-seq mapping rate against these assemblies using HISAT2 [24]. The RNA-seq mapping rates for the reference, the short-read-assembly, ABrujin-assembly, ABrujin-polished-assembly, Miniasm-assembly, Canu-assembly, SPAdes-hybrid-assembly, and MaSuRCA-assembly were 90.32%, 95.81%, 23.08%, 56.81%, 18.68%, 5.74%, 96.25%, and 95.06% (Table 1). The reason why the RNA-seq mapping rates for the short-read-assembly and hybrid assemblies were higher than those for the reference assembly is because the number of Ns per 100 kbp of reference was large and the same sequencing error profile of HiSeq gives raised sequence identity. For the assemblies using only long reads, the RNA-seq mapping rate was quite low, as the sequencing accuracy of MinION is low. In addition, the RNA-seq mapping rate of the ABrujin-polished-assembly, whose percent sequence identity to the reference was low even after polishing, suggests that this assembly cannot be sufficiently improved by polishing and is not suitable for gene expression analysis.

**Fig. 3:**
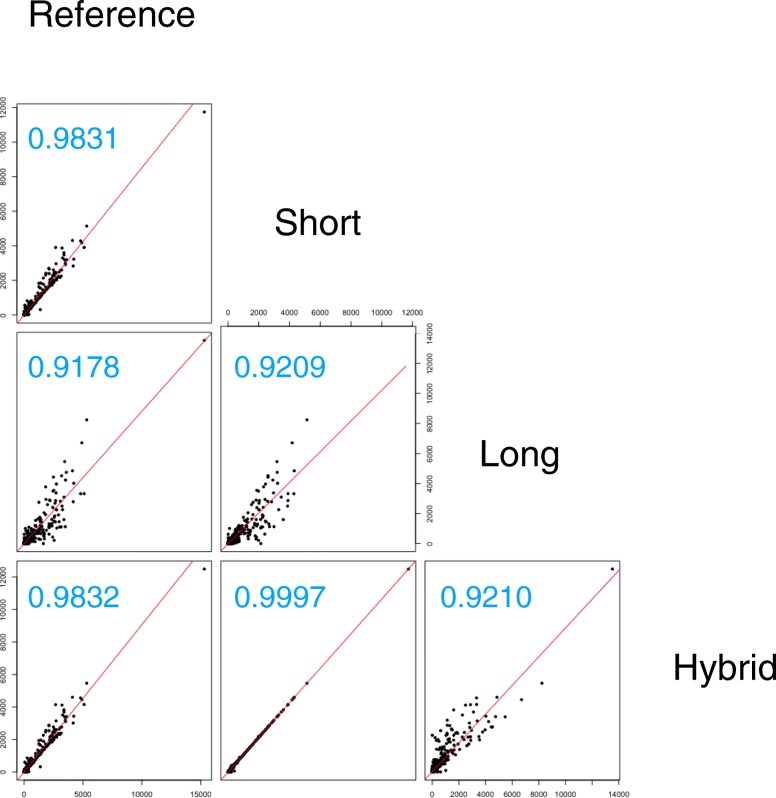
Correlation analysis of the gene expression level among four assemblies. Scatter plots demonstrates the TPM of single-copy-orthologs between all pair-wise assemblies. Each dot corresponds to a single gene. The red lines show regression line. The blue digits on the top right indicate the correlation coefficient value between each assembly. The Reference, Short, Long, and Hybrid show reference assembly previously published, short-read-assembly, ABrujin-polished-assembly, and MaSuRCA-assembly, respectively

We also investigated how much the gene expression levels of these assemblies differed from each other, which reflects the quality of the short-read-assembly, ABrujin-polished-assembly, and MaSuRCA-assembly. To obtain the same genes from these assemblies, we predicted orthologs and extracted 5343 single-copy orthologs suitable for comparing gene expression levels without the effects of paralogous genes. We then calculated the transcripts per kilobase million (TPM) [25] of these single-copy orthologs in each assembly. Finally, we evaluated the correlations between gene expression levels using scatterplots and correlation testing. The correlation coefficient between the short-read-assembly and MaSuRCA-assembly was the highest (0.9997) among all combinations, followed by the reference and MaSuRCA-assembly (0.9832) and the reference and short-read-assembly (0.9831) (Fig. 3). By contrast, the correlation coefficients of the ABrujin-polished-assembly using only long leads were the lowest of all assemblies (vs. reference 0.9178, short-read-assembly 0.9209, and MaSuRCA-assembly 0.9210) (Fig. 3). Differences in the correlation coefficients occurred for the same reason as in the mapping rates. The assembly using only long reads was not suitable for gene expression analysis as TPM greatly varied.

### Establishment of a de novo assembly pipeline for middle-sized genomes using MinION

The quality of assembly using only long reads was lower than that for other assemblies mainly because of the low sequencing accuracy, which is a restriction of the current nanopore technology. Consequently, it was not suitable for comparative genomics and transcriptomics as it reduces the precision of gene prediction and the RNA-seq mapping rate. Furthermore, these indices were not improved by polishing, indicating that assembling a high-quality genome of *C. variabilis* with only 56X MinION reads data is difficult. However, this problem was remedied by hybrid assembly using short reads; we established a pipeline for de novo assembly of middle-sized genomes using MinION long reads with Illumina short reads using the MaSuRCA-assembly.

**Fig. 4:**
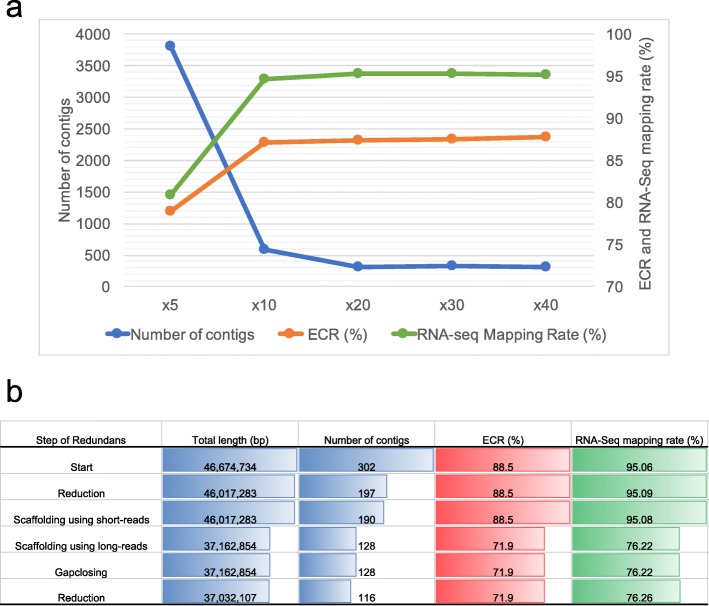
Optimization of an assembly pipeline by changing genome coverage and by reducing contigs. **a** Evaluation of assemblies varing read depth. The blue line shows number of contigs, the orange line shows ECR, and the green line shows RNA-seq mapping rate. The x-axis indicates read depth. **b** Examination of optimal conditions of Redundans for improving MaSuRCA-assemblies. The first column represents each step of Redundans pipeline. Each column represents index for evaluating these assemblies

As there are many target species of symbiotic algae for comparative genomic research, it is worthwhile to identify a better sequencing strategy with lower sequence read coverages. In addition, preparing massive and high-quality genomic DNA for these species that is long but fragmented enough to sequence using MinION is difficult with respect to culturing [26], and finding a smaller amount of input genomic DNA is critical for a better sequencing strategy. Therefore, we investigated the amount of MinION data needed to assemble a high-quality genome by changing the total sequence reads from *C. variabilis*, such as 5×, 10×, 20×, 30×, and 40× MinION read data. We then estimated the amount of MinION data required to establish a pipeline by evaluating these assemblies’ varying read depths using indices such as the number of contigs, ECR, and RNA-seq mapping rate. For assemblies using 5X data, the number of contigs, ECR, and RNA-seq mapping rate were 3809, 78.9%, and 80.87%, respectively (Fig. 4a). Using 10X, the number of contigs was obviously reduced, whereas the ECR and RNA-seq mapping rate were similar to those for the MaSuRCA-assembly (Fig. 4a). For assemblies using 20X data, all indices were similar to those for the MaSuRCA-assembly (Fig. 4a). A possible reason for the lack of improvement in the assembly indices after 10–20× is the sequence differences between the error-prone MinION raw reads and short reads. Given these results, we suggest that the high-quality genome is assembled using MinION read data of 10–20X the genome size.

**Fig. 5:**
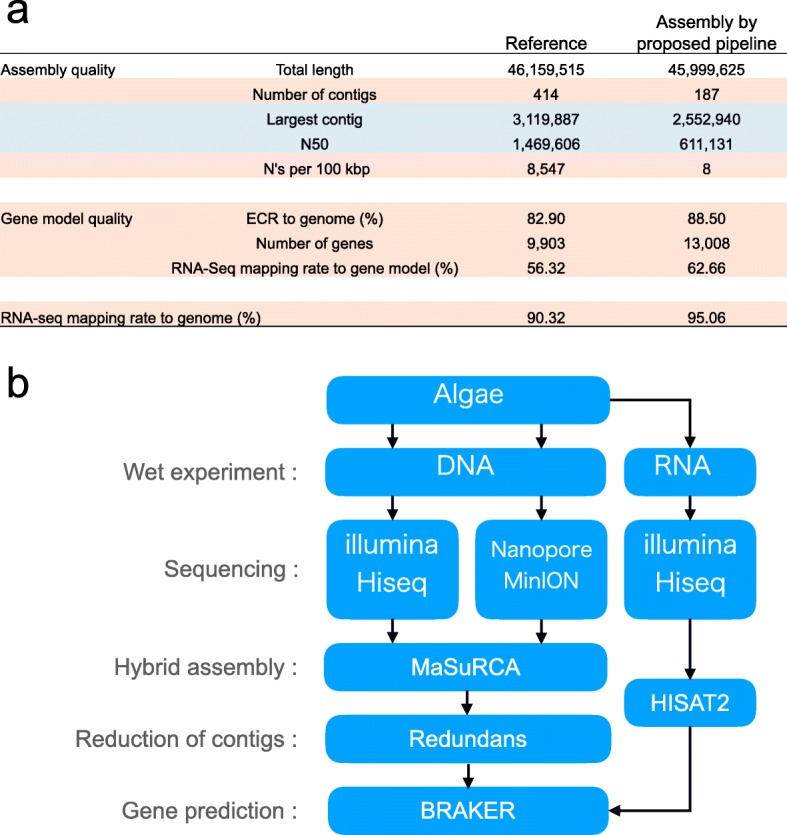
Overview of this study. **a **Comparison between reference (Reference) and most high-quality assembly (Assembly) in this study. Assebly by proposed pipeline generated by hybrid assembly using MaSuRCA, and then brushing up by Redundans. Improved indexes compared to reference are red colored, in contrast indexes which did not improved compared to reference are blue. **b** Overview of constructed pipeline of de novo assembly for middle-sized genome using MinION

Next, to reduce the number of contigs, we performed scaffolding MaSuRCA-assembly using Redundans [27], which reduces the redundant contigs and performs scaffolding. We examined the optimal conditions of Redundans by checking the assembly after each step of the Redundans pipeline using indices such as total length, number of contigs, ECR, and RNA-seq mapping rate. We found that the total length was about 10 Mbps shorter than the reference assembly and that the ECR and RNA-seq mapping rates were drastically reduced after the scaffolding step using long reads (Fig. 4b). For Redundans without scaffolding using long reads, the number of contigs, the largest contig, N50, the reference coverage, and the percent sequence identity to the reference were 187, 2,552,940 bp, 611,131 bp, 99.32%, and 95.58%, respectively, demonstrating that the ECR and RNA-seq mapping rates were the same as the originals (Fig. 5a). A possible reason for the reduction in assembly indices after scaffolding with long reads is sequence differences between the error-prone MinION raw reads and the MaSuRCA-assembly corrected by the short reads. In summary, we adopted Redundans without a scaffolding step using long read to brush up the pipeline, as this resulted in a reduced number of contigs.

**Fig. 6:**
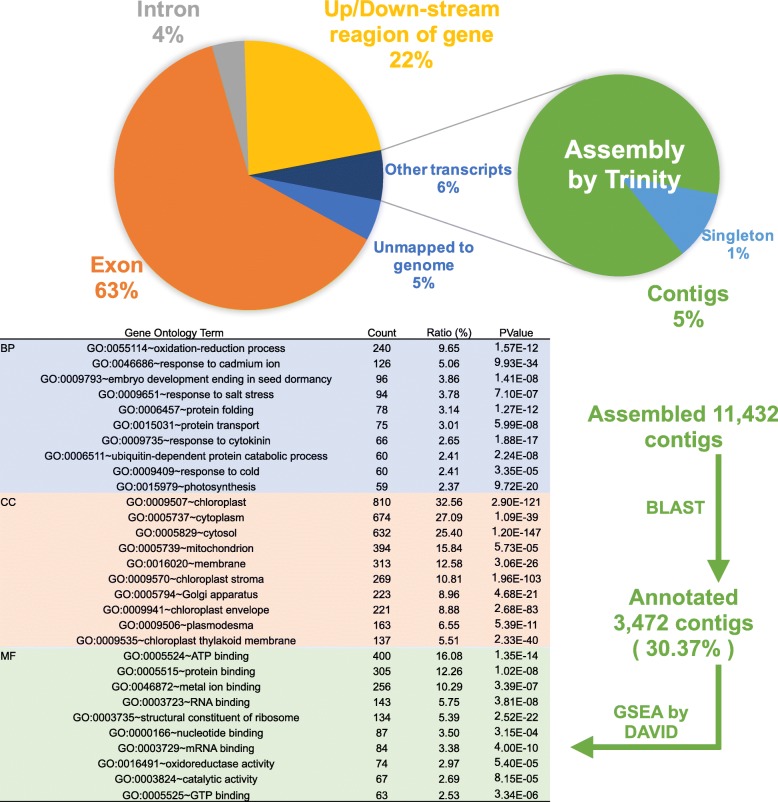
Analysis of what RNA-seq reads could not be mapped to the gene model. The left pie chart indicates where RNA-seq reads mapped to in the genome. The exon (orange), intron (gray), and up/down-stream (yellow) are ones that mapped to the predicted gene model. The other transcripts (dark blue) represents unmapped reads to the gene model but mapped to the genome. The unmapped to genome represents unmapped reads to the genome (light blue). The right pie chart represents proportion between not used reads (singleton) and used reads (contigs) in RNA assembly using reads of other transcripts. The bottom left figure shows result of GSEA analysis, BP (blue) shows biological process, CC (red) shows celluar component, and MF (green) shows molecular function

The next step was gene prediction from the assembly using BRAKER1 [28], a pipeline for unsupervised RNA-seq-based gene prediction. Using this method, we found 13,008 genes (Fig. 5a). The ECR of this gene model was about 10% higher than that of the gene model (reference), indicating that this gene model was more precise (Fig. 5a). To assess the comprehensiveness of this gene model, we examined how much RNA-seq could be mapped to the reference and current gene models. The RNA-seq mapping rates to the gene model (reference) and predicted gene model were 56.32% and 62.66%, respectively (Fig. 5a). This result indicates that the RNA-seq mapping rate was more precise than the gene model (reference) for predicting the gene model. However, we also found unmapped RNA-seq reads to the current gene model, as there was a discrepancy of about 30% between the RNA-seq mapping rate to the gene model using BRAKER1 and mapping rate to the assembled genome. We hypothesized that the unmapped reads might be derived from transcripts from predicted gene-related regions, such as introns, and the upstream and downstream regions of this gene model. We then aligned the RNA-seq reads to the region related to the gene model and found that 3.92% of RNA-seq reads mapped to the intron region and 22.50% mapped to the upstream and downstream regions (Fig. 6). These results supported our hypothesis, as 89.08% of RNA-seq reads were mapped to this gene model, including the intron and upstream and downstream regions. These results also indicate that untranslated regions might be predicted using RNA-seq. Moreover, we found that 6% of RNA-seq reads could not be mapped to the gene model as other transcripts. To investigate what these reads were, we constructed de novo RNA assembly annotated against *Arabidopsis thaliana* by means of a BLAST search. From the gene set enrichment analysis (GSEA) using gene ontology (GO) of annotated contigs, the following GO terms were enriched: in the biological process (BP), oxidation-reduction process (GO:0055114) and photosynthesis (GO:0015979); in the cell component (CC), chloroplast (GO:0009507), and mitochondrion (GO:0005739), etc. (Fig. 6). These results suggest that genes coded in the genome of mitochondria and chloroplasts might be not precisely predicted because BRAKER1 software predicts eukaryote genes, and these are derived from prokaryotes [28].

For the pipeline of de novo assembly of middle-sized genomes using MinION, a high-quality genome could be assembled by MaSuRCA using 80X coverage of short reads and 10–20X MinION reads. This assembly could be brushed up by Redundans without the scaffolding step using long read to reduce contigs. Finally, the genes were precisely predicted by BRAKER1 using RNA-seq (Fig. 5b). For the genome of *C. variabilis* assembled using this pipeline, the number of contigs and N were lower, but the largest contig and N50 were shorter than those for the reference using Sanger sequences with BAC clones (Fig. 5a). The number of contigs over 50 kbp was larger than that in the reference, indicating that each contig was longer on average. Our assembly is useful for analysis of horizontal gene transfer and synteny (Additional file 1: Figure S2).

## Conclusions

In this study, we sequenced *C. variabilis* using MinION and assembled genomes under various conditions. We then evaluated these assemblies in detail using assembly quality and alignment to reference as well as gene model quality and RNA-seq mapping rate. Evaluation of these assemblies revealed that the assembly with MinION reads only was not useful for comparative genomics and transcriptomics, as the percent sequence identity to the reference, gene prediction accuracy, and RNA-seq mapping rate were low. The cause of these low values was the low sequencing accuracy of MinION. However, this problem was resolved by hybrid assembly using the MaSuRCA assembler, and we then assembled a high-quality genome at levels comparable with the reference assembly and predicted a more accurate gene model. By this assembly, we established the pipeline of de novo assembly for middle-sized genome using MinION. The genomic change during the early stages of plastid acquisition can now be revealed by sequencing and comparing many algal genomes. Moreover, this pipeline offers a solution for the assembly of various middle-sized eukaryotic genomes with high-quality and ease.

## Methods

### DNA & RNA preparation

*Chlorella variabilis* strain NC64A (ATCC 50258) was cultured under illumination by a light-emitting diode lamp (14 h/10 h light/dark cycle) at room temperature (around 25 °C). We used 20% Gamborg’s B5 basal medium with mineral organics (Sigma-Aldrich, MO, USA) at pH 7.5 (referred to as 1/5 G medium), with 0.02% l-serine added (1/5 G + Serin) as the culture medium. Under these culture conditions, *Chlorella* cells in the stationary phase were collected by centrifugation for DNA and RNA extractions.

DNA extraction was performed using the DNeasy Plant Mini Kit (Qiagen, Düsseldorf, Germany) with modified cell fracturing. Fifty to one hundred μg of cells were incubated for 5 min in 400 μL Buffer AP1 with 4 μL RNase A at 65 °C. After adding 400 μL of glass beads (ø 0.1 mm), each sample was homogenized in BeadSmash 12 (WakenBTech, Kyoto, Japan) at 5000 rpm for 30 s. Homogenization was repeated five times, and then each sample was again incubated for 10 min at 65 °C. Subsequent procedures were performed according to the manufacturer’s directions.

For RNA extraction, the collected *Chlorella* cells were once reserved in 10-fold the volume of RNA*later* Stabilization Solution (ThermoFisher, MA, USA). After adding 400 μL of glass beads to each 400 μL solution, homogenization was performed as in the method for DNA extraction. We then added 500 μL of Buffer RLT (RNeasy Mini Kit, Qiagen) and vortexed and incubated the samples at 56 °C for 3 min. After centrifugation at 13,000 *g*, the supernatant was transferred to a new tube. Subsequent procedures were performed according to the directions of the RNeasy Mini Kit.

### Illumina library preparation and sequencing

Extracted genomic DNA and total RNA of *C. variabilis* were shipped to BGI-Shenzhen (Shenzhen, China) for library preparation and sequencing. For the DNA-seq and RNA-seq datasets, we sequenced genomic DNA and total RNA of *C. variabilis* using HiSeq 4000 and obtained 2 × 100 bp 35,007,584 and 25,396,225 paired-end reads, respectively.

### MinION library preparation and sequencing

Genomic DNA (6.4 μg) was fragmented to approximately 8 kbp using Covaris g-TUBE (Covaris, Woburn, USA). After purification using 0.4× AMPure XP beads (Beckman Coulter Inc., Brea, CA, USA) without a DNA repair step, the sequencing library was prepared using the SQK-LSK108 sequencing kit (Oxford Nanopore Technologies, Oxford, UK) following the manufacturer’s protocol (1D Genomic DNA by ligation). The sequencing library was loaded to R9 chemistry flowcell (FLO-MIN106) and sequenced with live base calling using MinKNOW for 48 h. FASTA and FASTQ files and the sequencing summary were generated using Poretools [29].

### De novo genome assembly and evaluation

The reference genome of *C. variabilis* (unmasked ChlNC64A_1_nuclear_scaffolds.fasta) was downloaded from the JGI Genome Portal [30]. After quality control using AfterQC [31], short reads were derived from HiSeq assembled by SPAdes v3.10.1 [14] with the default parameters. All MinION reads were assembled using ABrujin v1.0 [17] with parameters: -o 3000, Miniasm v.0.2 [18] pipeline with default parameters and Canu v1.5 [19] with default parameters. Hybrid assembly was performed using SPAdes v3.10.1 [14] with parameters: --nanopore and MaSuRCA v3.2.2 [20] with default parameters. The ABrujin-assembly was polished using Racon [21] and Nanopolish [22] using the base called data with Albacore v3.0.0 [32]. The indices of assembly quality were evaluated by Quast [33]. The reference coverage and percent sequence identity to the reference were calculated using Minimap2 v2.6 [34] with parameters: -ax asm5. The reference coverage was calculated from “the number of bases in mapped sequences” derived from Minimap2 [34] divided by the size of the reference genome. Similarly, the percent sequence identity to the reference was calculated from “the number of mapped bases” [34] divided by the size of the reference genome.

### Gene prediction accuracy and RNA-seq mapping rate

To evaluate gene prediction accuracy, the ECR of the assembly was calculated as the ratio of core genes whose full lengths were expected, including duplicated core genes using BUSCO with Eucaryota odb9 datasets (303 single-copy orthologs) [23]. The reference gene model of *C. variabilis* was Chlorella_NC64A.best_genes.gff deposited in the JGI Genome Portal [30]. The gene model was predicted using the BRAKER1 [28] pipeline with the default settings and evaluated using indices of the number, average length, and ECR generated by an R script and BUSCO [23]. The reads of the RNA-seq were mapped to the assembled genome using HISAT2 [24] with default settings, and then TPM [25] was calculated using StringTie [35]. To obtain the same genes from the reference, short-read-assembly, ABrujin-polished-assembly, and MaSuRCA-assembly orthologs were predicted using Orthofinder [36] and the single-copy orthologs were extracted. The correlation coefficient and scatterplot of TPM of extracted orthologs were generated by Python and R scripts.

### Establishment of pipeline

To examine MinION read depth for the hybrid assembly, genomes of *C. variabilis* were assembled using the MaSuRCA-assembler with 5×, 10×, 20×, 30×, and 40× MinION data, respectively. Next, these assemblies were evaluated on the basis of the number of contigs, ECR, and RNA-seq mapping rate. The optimal conditions of Redundans were examined by assessing the total length, number of contigs, ECR, and RNA-seq mapping rate for each genome assembly after the first reduction, scaffolding with short reads, scaffolding with long reads, gap closing, and final reduction steps of the Redundans pipeline, respectively. To use BRAKER1, we evaluated the gene model predicted from the brushed up genome. RNA-seq was mapped to this gene model and the reference gene model. Next, the mapping rates were calculated and compared. Furthermore, to characterize the unmapped reads, RNA-seq data were mapped to the intron and the upstream and downstream regions of the predicted gene model. The mapping rate for the predicted gene-related region was also calculated. Lastly, the upstream and downstream region was extracted from peripheral 1 kbp sequences for each predicted gene.

### Analysis of unmapped reads to the gene model

Unmapped RNA-seq reads to the gene model were assembled using Trinity v2.4.0 [37], and assembled contigs were then annotated using BLASTp with an e-value threshold of 10^− 5^ to the *A. thaliana* reference proteome data (UniProt Proteome ID: UP000006548). This blast result was analyzed by GSEA using DAVID [38] and the top 10 most frequent GO terms in the BP, CC, and MF category.

## Additional files

Additional file 1: **Figure S1.** Histogram of the length of raw reads derived form MinION. Much short reads under 500 bp were removed by means of purification using 0.4× magnetic beads before library preparation. **Figure S2.** The contigs of assembled genomes of *C. variabilis* sorted in descending order of the length. Reference were previously assembled genome, assembly were assembled genome using the pipeline constructed in this study. (PDF 95 kb)

Additional file 2: **Table S1.** Summary of sequencing using MinION. (XLSX 9 kb)

## Abbreviations

Core gene: 303 eukaryotic common single-copy-orthologs
ECR: Eukaryotic common gene conservation rate
GO: Gene ontology
GSEA: Gene set enrichment analysis
