# Supplementary material for: De novo assembly of middle-sized genome using MinION and Illumina sequencers
Source: BMC Genomics. 2018 Sep 24;19:700. doi: 10.1186/s12864-018-5067-1 (PMC6154909; doi:10.1186/s12864-018-5067-1)
Supplement: Supplementary file 1 — Figure S1. Histogram of the length of raw reads derived form MinION. Much short reads under 500 bp were removed by means of purification using 0.4× magnetic beads before library preparation. Figure S2. The contigs of assembled genomes of C. variabilis sorted in descending order of the length. Reference were previously assembled genome, assembly were assembled genome using the pipeline constructed in this study. (PDF 95 kb) [file 12864_2018_5067_MOESM1_ESM.pdf]

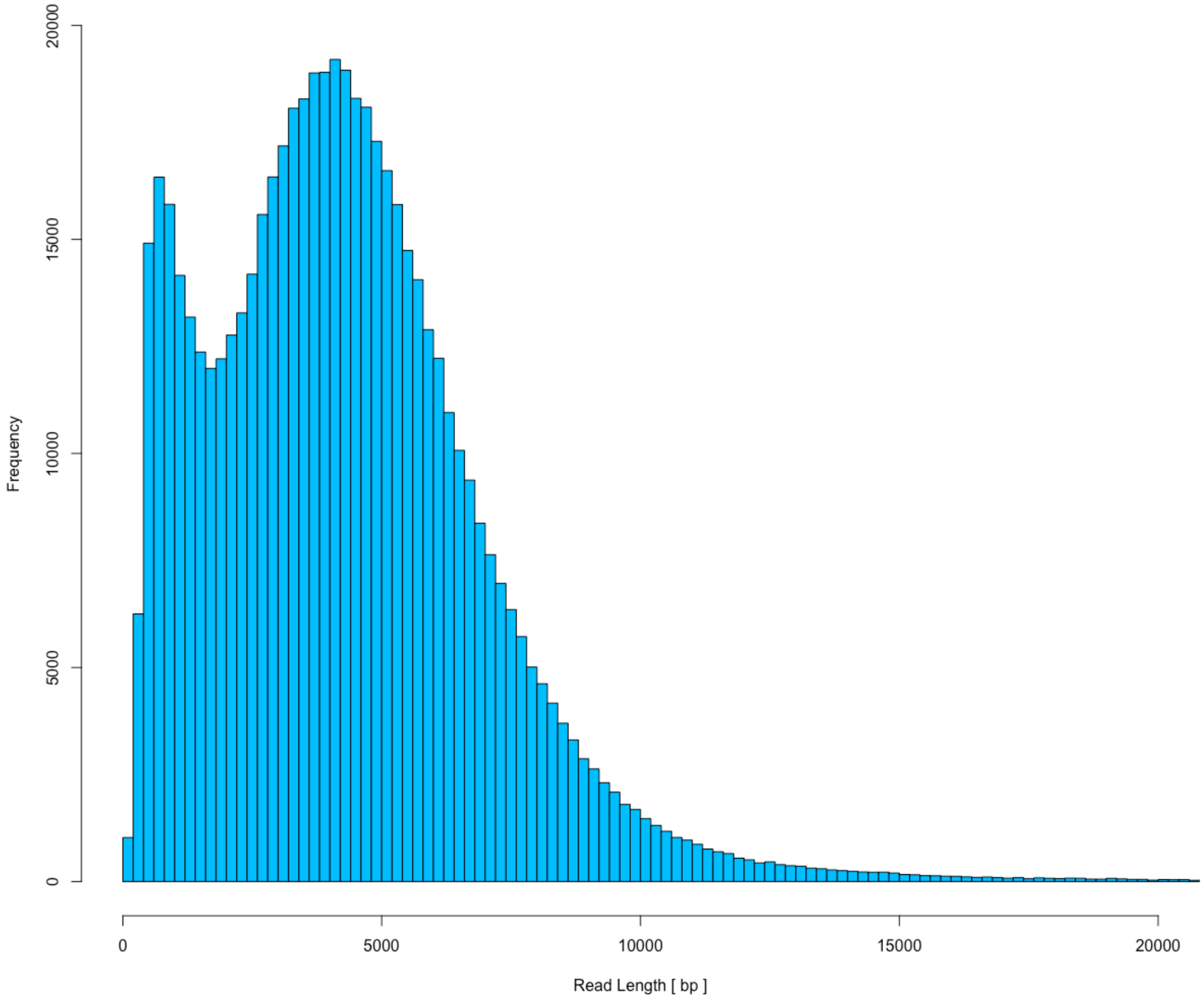

Figure S1. Histogram of the length of raw reads derived form MinION. Much short reads under 500 bp were removed by means of purification using 0.4x magnetic beads before library preparation.

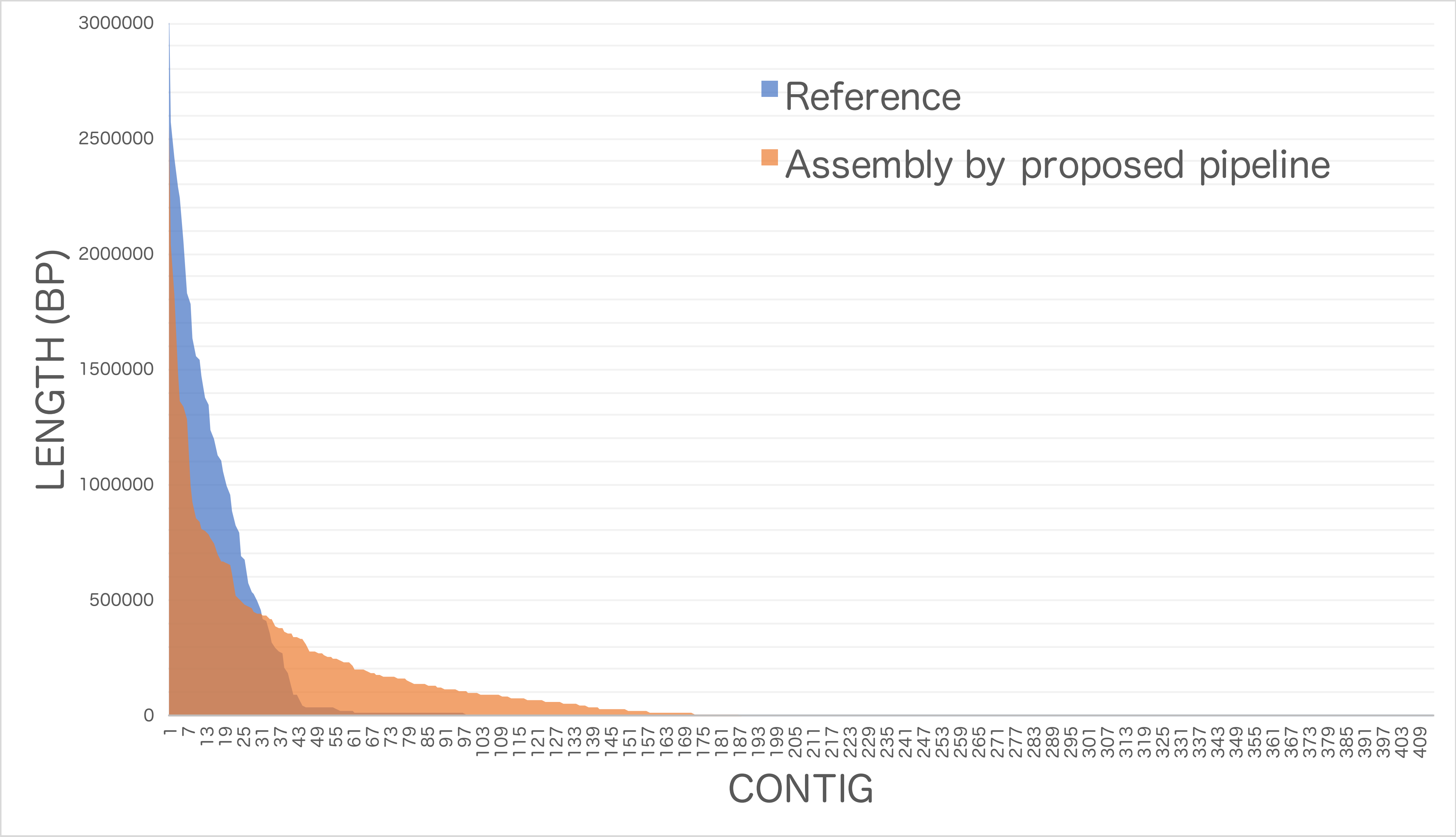

Figure S2. The contigs of assembled genomes of C. variabilis sorted in descending order of the length. Reference were previously assembled genome, assembly were assembled genome using the pipeline constructed in this study.
